# Differences in selected blood parameters between brachycephalic and non-brachycephalic dogs

**DOI:** 10.3389/fvets.2023.1166032

**Published:** 2023-08-15

**Authors:** Sandra Kämpf, Simone Fenk, Ankie Van Cromvoirt, Nikolay Bogdanov, Sonja Hartnack, Martina Stirn, Regina Hofmann-Lehmann, Iris Margaret Reichler, Anna Bogdanova

**Affiliations:** ^1^Red Blood Cell Research Group, Institute of Veterinary Physiology, Faculty of Vetsuisse, University of Zurich, Zürich, Switzerland; ^2^Center for Clinical Studies (ZKS), Vetsuisse Faculty, University of Zurich, Zürich, Switzerland; ^3^Clinic of Reproductive Medicine, Vetsuisse Faculty, University of Zurich, Zürich, Switzerland; ^4^Section of Epidemiology, Vetsuisse Faculty, University of Zurich, Zürich, Switzerland; ^5^Clinical Laboratory, Department for Clinical Diagnostics and Services, Vetsuisse Faculty, University of Zurich, Zürich, Switzerland

**Keywords:** dog, brachycephaly, brachycephalic obstructive airway syndrome, red blood cells, blood markers, stress, hypoxia

## Abstract

**Introduction:**

Cranial and upper-airway anatomy of short-nosed, flat-faced brachycephalic dogs predisposes them to brachycephalic obstructive airway syndrome (BOAS). Periodic apnoea increased inspiratory resistance, and an inability to thermoregulate effectively are characteristic of BOAS, but internationally accepted objective markers of BOAS severity are missing. The objective of this study was to compare the selected blood parameters between non-brachycephalic (NC) and brachycephalic (BC) dogs, exploring the possibility of developing a blood test for BOAS severity grading in the future.

**Methods:**

We evaluated blood biochemistry, complete blood cell counts, red blood cell (RBC) indices, reticulocyte counts, a blood-born marker of intermittent hypoxia (glutathione, NO production), RBC hydration, deformability, and blood markers of metabolic changes and stress between BC (*n* = 18) and NC (meso- and dolichocephalic, *n* = 22) dogs.

**Results:**

Reticulocyte counts and the abundance of middle-fluorescence immature reticulocytes were significantly (*p* < 0.05) higher in BC dogs compared to NC dogs. BC dogs had significantly more NO-derived NO${}_{2}^{-}$/NO${}_{3}^{-}$ in plasma than NC dogs. RBCs of BC dogs were shedding significantly more membrane, as follows from the intensity of eosin maleimide staining, and had a significantly higher mean corpuscular hemoglobin concentration than NC dogs. Intracellular reduced glutathione content in RBCs of BC dogs was significantly lower, while plasma lactate was significantly higher in BC dogs compared to NC dogs. Plasma cholesterol and triglycerides were significantly lower, and cortisol was significantly higher in BC dogs compared to NC dogs. Eosinophil counts were significantly lower and the neutrophil-to-lymphocyte ratio was higher in BC dogs compared to NC dogs.

**Discussion:**

Taken together, our findings suggest that the brachycephalic phenotype in dogs is associated with alterations at the level of blood cells and, systemically, with oxidation and metabolic changes. The parameters identified within this study should be further investigated for their potential as objective indicators for BOAS.

## 1. Introduction

For decades, short-nosed and flat-faced brachycephalic dog breeds have been in the top ten lists of the most popular breeds worldwide (1–3). Their neotenous appearance fitting the “baby scheme” with large eyes, bulging craniums, and retreating chins (4) as well as broadly advertised “low-maintenance” lifestyle with no need for long walks or physical activity (5) supported their popularity as a supposedly suitable companion dog in the city. Breeding for exaggerated foreshortening and/or flattening of the facial skeleton, the screw-like tail, and the stunted growth resulted in a variety of congenital functional or anatomical anomalies in various organ systems. The reduced health status of brachycephalic dogs compared to normocephalic dogs is well documented (6, 7). Numerous clinical signs are associated with the brachycephalic obstructive airway syndrome (BOAS) caused by the breed-phenotype specific anatomical changes of the upper airway (8–12).

Extreme phenotypes are presented with stenotic nares, excessive and aberrant nasopharyngeal conchae, a thickened and elongated soft palate, nasopharyngeal mucosal and tonsillar hypertrophy, a relative macroglossia, and a hypoplasia of the trachea, reducing air volume of the upper airways (11–13). Inspiratory resistance, already high at rest in brachycephalic dogs, grows further under certain circumstances (exercise or higher ambient temperature), increasing the risk of tracheal collapse (12). Higher negative intraluminal pressure is associated with inflammation, mucosal oedema, which may lead to tonsillar eversion, and everted laryngeal ventricles which could result in laryngeal or bronchial collapse (14). Furthermore, the chronic high negative intrathoracic pressure developed to overcome the high upper airway resistance is suggested to be the cause of gastrointestinal disease (15, 16). As thermoregulation in dogs depends largely on respiratory evaporative cooling within the upper respiratory tract, brachycephaly impacts not only the breathing pattern but also thermoregulation (17). Brachycephalic dogs die on average 3 years earlier than normocephalic or mesocephalic dogs of the same size, which is likely to be linked to the symptoms arising from BOAS (18, 19).

Correct assessment of severity of BOAS condition is a prerequisite to the targeted selective breeding for healthier phenotype in brachycephalic breeds. However, clear international guidelines are currently lacking for the BOAS severity grading. The parameters that are used by veterinarians include anatomical examination [cranial indices, computed tomography, and rhinoscopy (20–23)], respiratory performance [full-body barometric plethysmography (24–28)], physical performance [trotting test (28, 29)], and assessment of blood oxygenation [pulse oximetry and blood CO-oximetry (9, 30)]. Some of these procedures require skilled personnel, while others need expensive and unconventional equipment. Blood oxygenation is a volatile parameter that stays within a normal range when dogs are not exercising and/or overheated (14, 22, 29–32).

However, other more stable blood parameters may better reflect the intermittent hypoxic state (9, 22, 33) that is experienced by brachycephalic dogs (34). Those include the NO derivatives (nitrite and nitrate), pro-oxidative markers and pro-inflammatory cytokines, metabolic markers, and stress markers, including those of cardiac overload (35–39). Brachycephalic breeds show higher propensity to hypercoagulation (40). Together, these findings suggest that blood parameters could contribute to the identification of dogs affected by BOAS. Up to now the impact of brachycephalic phenotype on red blood cell (RBC) morphological heterogeneity, deformability and hydration state, redox state, and glycolytic activity, as well as on RBC turnover, has not been investigated.

The aim of this study was to answer the phase I research question “Do blood test results in brachycephalic dogs differ from those in non-brachycephalic ones?” in the context of diagnostic accuracy studies (41). Our list of the selected parameters of interest included the common red blood cell indices, complete blood cell count, and clinical blood biochemistry, as well as the parameters that were shown to be affected in blood of human patients with obstructive sleep apnea (OSA). They include plasma NO derivatives (42) and oxidative stress markers such as non-protein and total reduced thiols and methemoglobin (43). As the potential screening parameters should not be affected by blood sample transportation from the site of withdrawal (e.g., dog show) to the lab, we also investigated the tentative impact of shipment on some of the most informative parameters. The outlook of the present study and our long-standing goal is the establishment of a set of suitable screening parameters that objectively and reliably identify dogs suffering from BOAS and may be used alongside currently existing tests for BOAS grading (cranial anatomy, trotting test, and plethysmography).

## 2. Materials and methods

### 2.1. Study cohorts of non-brachycephalic and brachycephalic dogs

The Veterinary Department of the Canton of Zurich has approved the study protocol (ZH161/19) and the study was conducted according to Swiss law. In the prospective, blinded study 42 privately owned dogs were enrolled. Two dogs were subsequently excluded (see Section 3.1); thus, a total of 40 dogs were included in the study. Owners of the dogs were contacted personally. Prior to inclusion, all owners signed an informed consent form. Inclusion criteria for the dogs were: (i) at least 12 weeks of age; (ii) a body weight of at least 5 kg; (iii) not treated with glucocorticoids, non-steroidal drugs, or other medicaments that could affect the breathing for at least 2 months prior to the enrollment date; and (iv) clinically healthy according to owner's perception, except for clinical signs associated with BOAS. While assigning the dogs into the brachycephalic group, we relied on the breed-specific brachycephalic phenotype (7). Two pug mixed-breed dogs were additionally tested for cranial indices using several morphometric approaches [(10, 22, 44, 45), for details see Supplementary data] and assigned to the brachycephalic group. Veterinarians from the Clinic of Reproductive Medicine at the University Animal Hospital collected the necessary information on the enrolling dogs as well as 6 ml venous blood from *vena jugularis* or *vena cephalica*. Lithium-heparin and K_2_-EDTA were used as anticoagulants. The blood samples were numbered and anonymized before being transferred to the lab. Blood parameters for the freshly drawn samples were obtained for all 40 dogs. The first exploratory phase of the study employing 20 dogs (dogs #1–20 in Supplementary Table S1) revealed several experimental blood parameters that differed significantly between the brachycephalic (BC) dogs and non-brachycephalic control (NC) dogs. These parameters were tested for the possible changes related to 6 or 24 h of simulated shipment in a second phase involving 20 additional dogs (dogs # 21–26, 28–39, 41, 42 in Supplementary Table S1).

### 2.2. Blood biochemistry and hematology analyses

Blood samples were analyzed within hours after collection. Clinical blood biochemistry in heparinized blood samples and hematological analyses in the samples anticoagulated with K_2_-EDTA were performed using a Cobas ^®^ 6000 501 analyzer (Roche Diagnostics AG, Rotkreuz, Switzerland) and a Sysmex XN-1000V (Sysmex Suisse AG, Horgen, Switzerland), respectively. Plasma cortisol concentration was measured using an Immulite 2000 (Siemens Schweiz AG, Zurich, Switzerland). C-reactive protein (CRP) concentration in plasma of heparinized blood samples was determined using a canine-specific immunoturbidimetric assay (Randox cCRP; Randox Laboratories Ltd., Crumlin, UK) (46). The hematological parameters measured included complete blood cell count (CBC), reticulocyte count and maturity (low, middle, and high fluorescence), reticulocyte hemoglobin, and thrombocrit. Blood biochemistry was performed using heparin anticoagulated blood. Total protein, albumin, bilirubin, glucose, urea, creatinine, cholesterol, triglycerides, alkaline phosphatase, alanine aminotransferase, creatine kinase, lipase, lactate dehydrogenase, Na^+^, K^+^, Cl^−^, total Ca^2+^, and phosphate were assessed in plasma. Heparinized blood was centrifuged at 1862 × g for 5 min; plasma was subsequently collected, and one aliquot was directly analyzed. In addition, ionized Ca^2+^ was measured using ABL800 Flex blood analyzer (Radiometer RSCH GmbH, Thalwil, Switzerland).

### 2.3. CO-oximetry

The K_2_-EDTA blood samples were used for this assay within 20 min after withdrawal. Total hemoglobin blood content (Hb), as well as percentage of met-hemoglobin, oxy-hemoglobin, and carboxy-hemoglobin, were monitored using Avoximeter 4000 (Instrumentation Laboratory GmbH, Bedford, MA). Due to high reproducibility (data not shown), a single measurement per sample was performed for this assay.

### 2.4. Ektacytometry

RBC rheology was assessed within 2 h after blood collection using the osmoscan mode of Laser Optical Rotational Red Cell Analyzer (Lorrca MaxSis, RR Mechatronics) (47, 48). Two hundred microliters of K_2_-EDTA-anticoagulated whole blood were mixed with 5 ml of isotonic PVP solution and the elongation of RBCs under conditions of 30 Pa shear stress at 37°C was assessed as a function of extracellular osmolarities in 0–500 mOsm/kg range. The osmoscan curves were obtained and the following parameters calculated from these curves: elongation index (EI) at optimal osmolality (EImax), osmolality of the hypotonic solution at which 50% of cells lyse while exposed to shear (O_min), and the osmolality at which EI is reduced to a half of EImax under hypertonic conditions (O_hyper). Elongation index at the osmolarity O_min and that at the O_hyper osmolarity (EI_Omin and EI_Ohyper) were also detected along with the area under the osmoscan curve (Area). More information on the method and the parameters may be found elsewhere (48, 49).

### 2.5. Detection of membrane loss and intracellular reduced thiols in canine RBCs by flow cytometry

Eosine 5'-maleimide (EMA) was used to assess the abundance of anion exchanger AE1 in the membranes of RBCs in heparinized whole blood samples. This is a common test to assess membrane surface area and premature membrane loss in human patients with hereditary spherocytosis and other hereditary hemolytic anemias (50). A triple-set of samples was prepared as follows. Two microliters of heparinized blood were suspended in 1 ml of canine plasma-like medium (CPLM) containing, in mM, 165 NaCl, 4.6 KCl, 0.15 MgCl_2_, and 10 glucose 20 HEPES–Tris-OH, pH 7.40 at room temperature supplemented with 0.1 % Bovine serum albumin. Stock solution of EMA (Merck) was prepared by dissolving 10 mg of EMA in 40 μL of dimethyl sulfoxide (DMSO) and stored at −20°C for up to a month. A 50x solution was prepared fresh before the experiment by dissolving 2 μL of stock solution in 1 mL of CPLM. Fifty microliters of this 50x solution were added to 950 μL of CPLM, and 2 μL of blood were then added to the final 1 ml of the 1x EMA solution. The cells were allowed to bind to EMA for 1 h in the darkness at room temperature. Thereafter, the cells were spun for 30 seconds at 2500 × g, room temperature (Eppendorf centrifuge), the extracellular EMA was aspirated, and the cells were washed twice with CPLM and resuspended in it to the volume of 1 ml. Fluorescence of EMA was then estimated by flow cytometry (Gallios, Beckman Coulter) at 633 nm excitation – 660/20 nm emission wavelengths.

Total reduced thiol content of RBCs was detected in heparinized blood using monobromobimane (MBBr) as a label (51). Stock solution of 20 mM MBBr (ThermoFisher) was prepared on DMSO, stored at −20°C, and used at a final concentration of 10 μM. An aliquot of whole blood (1–2 μL) was incubated with the fluorescent probe for 1 h in the dark and the fluorescence intensity was detected by flow cytometry at 405 nm excitation – 420/50 nm emission wavelengths.

For both EMA and MBBr, fluorescent signal was recorded for 100 000 cells in each sample of triplicates, at medium flow rate. Fluorescence intensity forward and side scatter as well as their variance was estimated and used as an indirect marker of shape and its heterogeneity. Kaluza software (Beckman Coulter) was used for the analysis.

### 2.6. Intracellular GSH and GSSG measurements

Non-protein thiols, of which more than 90% were reduced glutathione (GSH) with the rest being largely Cysteine (52), were measured using Ellman's reagent as described earlier (53, 54). A 300 μl aliquot of EDTA-preserved canine whole blood was mixed with 1.2 ml of 5% (w/w) solution of trichloroacetic acid and left to react for 30 min on ice. Denatured hemoglobin and other proteins were precipitated by centrifugation at 10 000 × g for 10 min at 4°C. Supernatant was neutralized to pH 7–8 using concentrated Tris(hydroxymethyl)aminomethane solution.

Four aliquots of 100 μl of neutralized supernatant were mixed each with 900 μl of EDTA/Phosphate buffer containing 100 mM NaH_2_PO_4_ and 10 mM Na_2_-EDTA at pH 7.5. Baseline detection of absorbance at a wavelength of 412 nm was detected using Lambda 25 spectrophotometer (Perkin Elmer). Stock solution of 10 mM 5,5′-Dithio-bis-(2-nitrobenzoic acid) (DTNB, Ellman's reagent) was prepared on EDTA/Phosphate buffer. After this, 10 μl of this stock solution was added to the supernatant samples and allowed to react for 3 min at room temperature. Thereafter, absorbance at 412 nm was measured and baseline values subtracted.

A second set of supernatant samples was used for GSSG detection. In these samples GSSG was reduced to GSH. Reduction was catalyzed by glutathione reductase and NADPH served as a donor of electrons. Stock solutions in EDTA/Phosphate-buffer were prepared for type II crude glutathione reductase from wheat germ (Sigma, 0.05 U/mg, Lot 33H7009, 10 mg/ml) and for NADPH-Na salt (Sigma, 3.33 mg/ml). Thereafter, 100 μl of supernatant sample was mixed with 880 μl EDTA/Phosphate buffer and supplemented with 10 μl glutathione reductase and incubated for 12–15 min. Then 10 μl of NADPH stock solution was added and incubation was continued for a further 20 min. Thereafter, baseline absorption was assessed at 412 nm (Lambda 25 spectrophotometer, Perkin Elmer) and 10 μl of DTNB was added to assess the non-protein thiols as described above for GSH detection.

### 2.7. Detection of NO${}_{2}^{-}$ and NO${}_{3}^{-}$ in plasma

Chemiluminescence detection of NO was used for assessment of RNO (NO_2_- +NO_3_-) in canine plasma form heparinized blood samples (55). Plasma was collected after 4 min of centrifugation at 4000 × g, 4°C. Plasma color was visually controlled for free hemoglobin. When free hemoglobin was visible, the sample was discarded, as free hemoglobin may reduce NO_2_- to NO and result in underestimation of RNO levels. In order to detect plasma nitrate (NO${}_{3}^{-}$) concentration using chemiluminescence method, nitrate was converted to nitrite (NO${}_{2}^{-}$) using cadmium-copper-based reduction assay and then the concentration of NO${}_{2}^{-}$ was estimated as follows. A 50 μl bolus of the sample containing 1.65 g KI and 0.57 g I_2_ was injected into the acidic triiodide (I_3_) reagent (also known as Brown's solution), pre-heated to 65°C, and dissolved in a mixture of 15 ml ddH_2_O, and 200 ml glacial CH_3_COOH. Upon reduction of NO${}_{2}^{-}$ to NO in the reaction chamber, which was purged by the gas NO, the sample was delivered to the CLD-88 analyzer (ECO MEDICS, Durnten, Switzerland). Interaction NO with O_3_ resulted in light emission that was sensed by the chemiluminescence detectors. The resulting signal was processed using PowerChrom 280 system (eDAQ Pty; Spechbach, Germany) and analyzed using eDAQ Chart v. 5.2.9 software.

### 2.8. Shipment simulation study

Blood samples from 20 dogs (#21–#42, Supplementary Table S1) were stored shaken on an orbital shaker RS-LS 10 (Phoenix Instruments, Garbsen, Germany) at 200 rotations per min and analyzed within the first 30 min after collection (To), and then 6 h and 24 h after the simulated shipment. The parameters that were tested for their stability in time included plasma lactate (detected with Lactate Pro, Axon Lab AG, Baden,) glucose (Glucose monitor Contur XT, Ascensia Diabetes Care Holdings AG), EMA staining, and intraerythrocytic GSH content.

### 2.9. Statistics

Statistical analysis was performed using the R software (56). Shapiro-Wilk test was used to check whether the data were normally distributed. Subsequently, a parametric (student's *t*-test) or non-parametric test (Wilcoxon test) was used to compare the two experimental groups. Differences were found to be statistically significant when *p* < 0.05. Parameters, for which this condition applied, were addressed to as “different” between the BC and NC groups in “Results” and “Discussion” sections. Specific details and the *p*-values can be found in figure legends. Values are shown as boxplots with the minimum, the maximum, the sample median, and the lower-upper interquartile ranges. Since the seven parameters indicative for the RBC indices and the eight for white blood cells and platelets were measured in the same blood samples, they cannot be considered independent. This lack of independence may induce multiplicity, leading to a too small *p*-value and possibly spurious significant results. Therefore, as a note of caution, in our exploratory approach, we also applied other machine learning approaches to assess if the same variables (blood parameters) are consistently found different between NC and BC dog groups. First, we used the function varrank available in the varrank package (57) with a forward algorithm and the method Estevez. This function sequentially compares the relevancy/redundancy balance of information across a set of variables. Second, we constructed a learning vector quantization model based on a 10-fold cross-validation with the packages caret (58) and e1701 (59) to rank the variables by importance in a model-free approach. Third, we used the function Boruta (60) to search for the most relevant variable by random forest. Fourth, another random-forest approach to perform variable ranking was used with the package varSelRf (61) with 200 trees.

## 3. Results

### 3.1. Study participants' characteristics

The cohort of BC dogs included 16 purebred dogs belonging to known brachycephalic breeds (7, 62, 63) and two pug mixed-breed dogs with cephalic indices as well as craniofacial ratios characteristic for brachycephalic breeds (6, 22, 64). Their age ranged from 0.5 to 12 years; five were males (one castrated) and 13 females (five spayed) (Supplementary Table S1). The weight of BC dogs spanned from 6 kg to 40 kg; most of the animals in this group (*n* = 11) were French Bulldogs. The cohort of NC dogs included 22 purebred and mixed breed dogs of 0.5–12.5 years of age, from large (31 kg) to small size (6 kg), of which 17 were females (nine spayed) and five males (one castrated). On average, body weight of the BC group was significantly lower compared to the NC group [mean 13.2 kg (median 10.3 ± 8.6 and mean 19.7 kg) (median 19.9 ± 8.8, respectively; *P* = 0.016]. None of the dogs showed adverse events during the blood withdrawal. Two dogs that had been enrolled in the BC group (#27, an American Pitbull, and #40, a Boxer-Dalmatian mix) were subsequently excluded due to missing clear characteristics of BC dogs. They are therefore not listed in the Supplementary Table S1.

Raw data that were obtained in our study may be found at the open-source repository Zenodo platform (doi: 10.5281/zenodo.7510523).

### 3.2. Hypoxic markers, RBC production, and turnover markers

**Figure 1:**
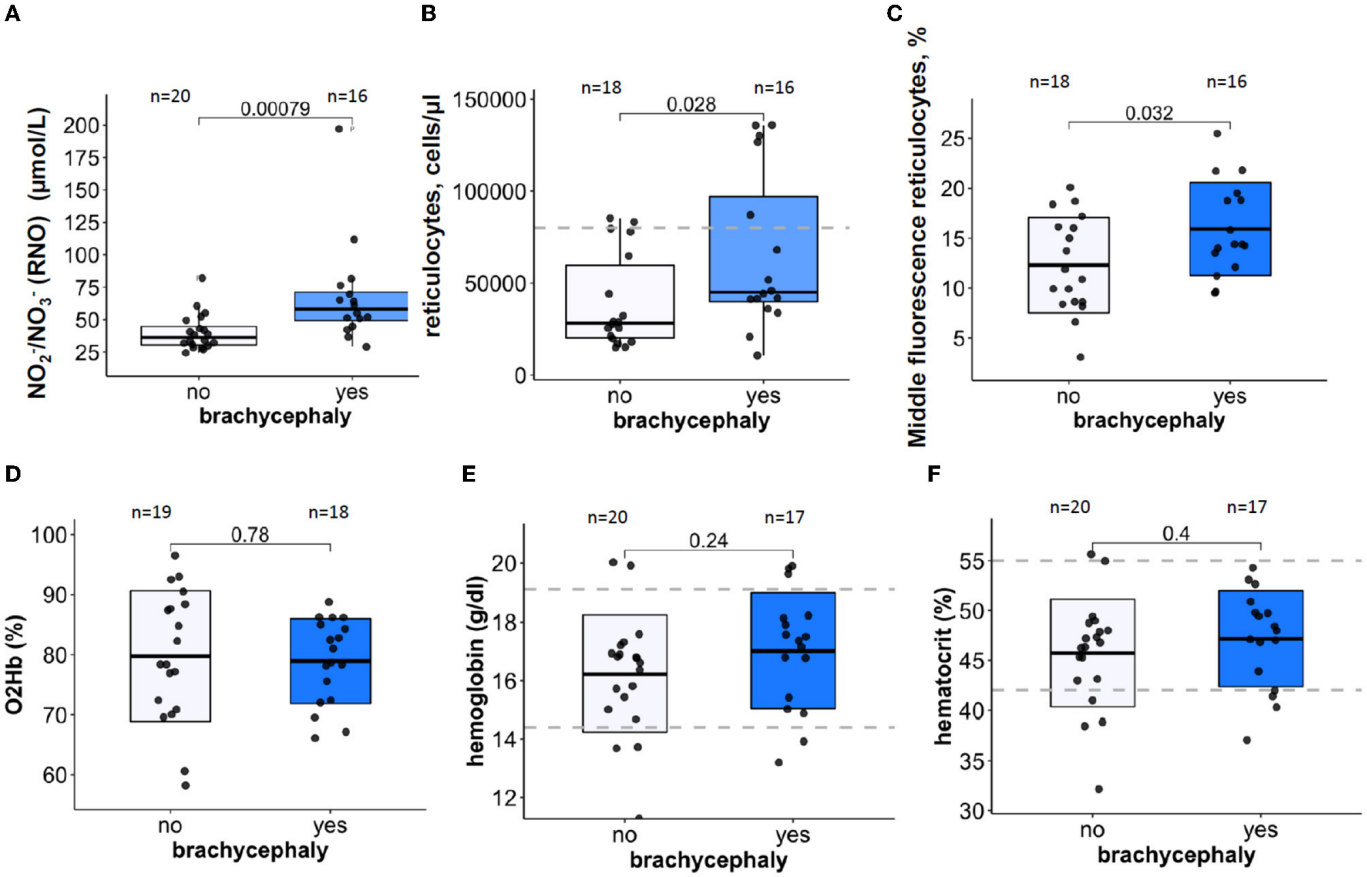
Oxygen-sensitive parameters in blood samples of brachycephalic (BC) and non-brachycephalic (NC) dogs shown as box-and-whiskers plots. Plasma levels of nitrite and nitrate RNO **(A)**, reticulocytes count **(B)**, Middle fluorescence intensity reticulocyte counts in % **(C)** Hemoglobin O_2_ saturation in mixed venous blood **(D)**, hemoglobin **(E)**, and hematocrit **(F)**. After the normality of distribution of the data was tested, Unpaired *t*-test or the Wilcoxon signed-rank test was performed to detect significance of differences between the BC and NC groups. *P*-values are shown for each parameter. Numbers above the bars are the number of study participants. Blue boxes are for the brachycephalic group and white ones for the non-brachycephalic group. Dotted lines indicate a healthy reference range.

The BC dogs showed significantly higher plasma concentrations of NO-derivatives nitrite and nitrate (RNO) (Figure 1A) and an increase in reticulocyte counts (Figure 1B) compared to the NC dogs. Apart from that, the reticulocytes with medium fluorescence intensity (MFI) were more abundant in the BC group than in the NC group (Figure 1C). No differences were observed in the low (LFI) and high fluorescence intensity (HFI) of the reticulocytes (see raw dataset). Other acute (O_2_ saturation of hemoglobin, Figure 1D) and chronic (Hb and Hct, Figures 1E, F) markers of hypoxia did not differ between the groups.

**Figure 2:**
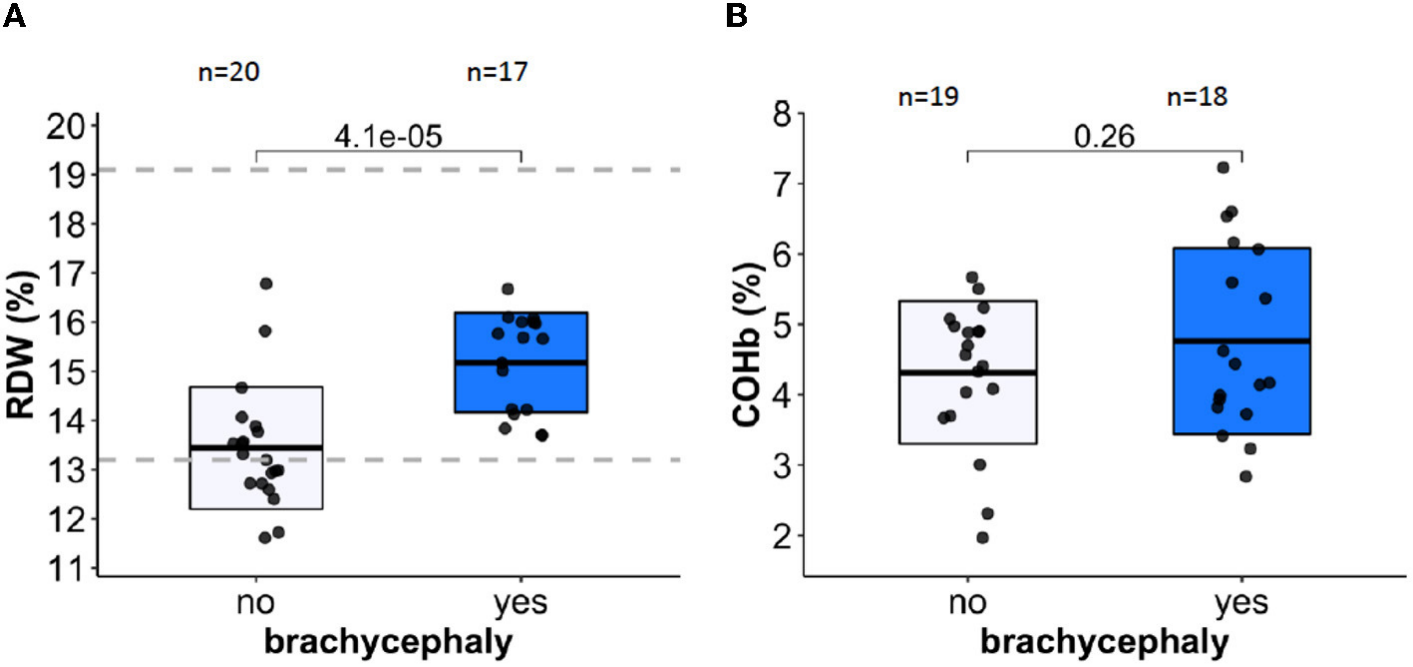
Red blood cell distribution width (RDW) and carboxy-hemoglobin abundance are shown in panels **(A, B)** respectively as markers of RBC distress and hemolytic activity. Dotted lines indicate healthy reference range.

Red blood cell distribution width (RDW) showed statistically significant difference between the BC and NC groups (Figure 2A, Table 1, and Supplementary Figures 1–3). This finding was supported using both common (*t*-test, Wilcoxon) statistical approaches as well as three machine-learning approaches (Supplementary Figures 1–3), indicating that this is an important variable to predict the “BC-specific blood phenotype”. No signs of even mild hemolytic activity was detected in any of the groups. CO-Hb (Figure 2B) and bilirubin (lower than 2.5 μM for both groups) were not higher in the BC dogs compared to the NC dogs.

**Table 1:** Red blood cell indices for non-brachycephalic (*n* = 22) and brachycephalic dogs (*n* = 18).

**Parameter**	**Unit**	**Reference range clinics**	**BC group (*n =* 18)**	**SD**	**NC group (*n =* 22)**	***SD***	P^*^
RBC count	x10^6^/μL	6.1–8.1	6.89	0.77	6.78	0.83	0.69
Hb	g/dl	14.4–19.1	17.01	1.97	16.23	2.00	0.24
Hct	%	42–55	47.16	4.80	45.73	5.39	0.4
MCV	fl	64–73	68.59	2.71	67.51	1.92	0.18
MCHC	g/dl	34–36	36.02	0.74	35.52	0.58	**0.03**
MCH	pg	23–26	24.72	1.20	23.94	0.78	**0.03**
RDW	%	13.2–19.1	15.18	1.01	13.44	1.24	**0.000041**

^*^P-values here are not adjusted for multiplicity. Highlighted in bold are the P values below 0.05. Hb, hemoglobin; Hct, hematocrit; MCV, mean corpuscular volume; MCHC, mean corpuscular hemoglobin concentration; MCH, mean corpuscular hemoglobin; RDW, red blood cell distribution width. All the variables in this table are normally distributed.

### 3.3. Red blood cell membrane stability and hydration

**Figure 3:**
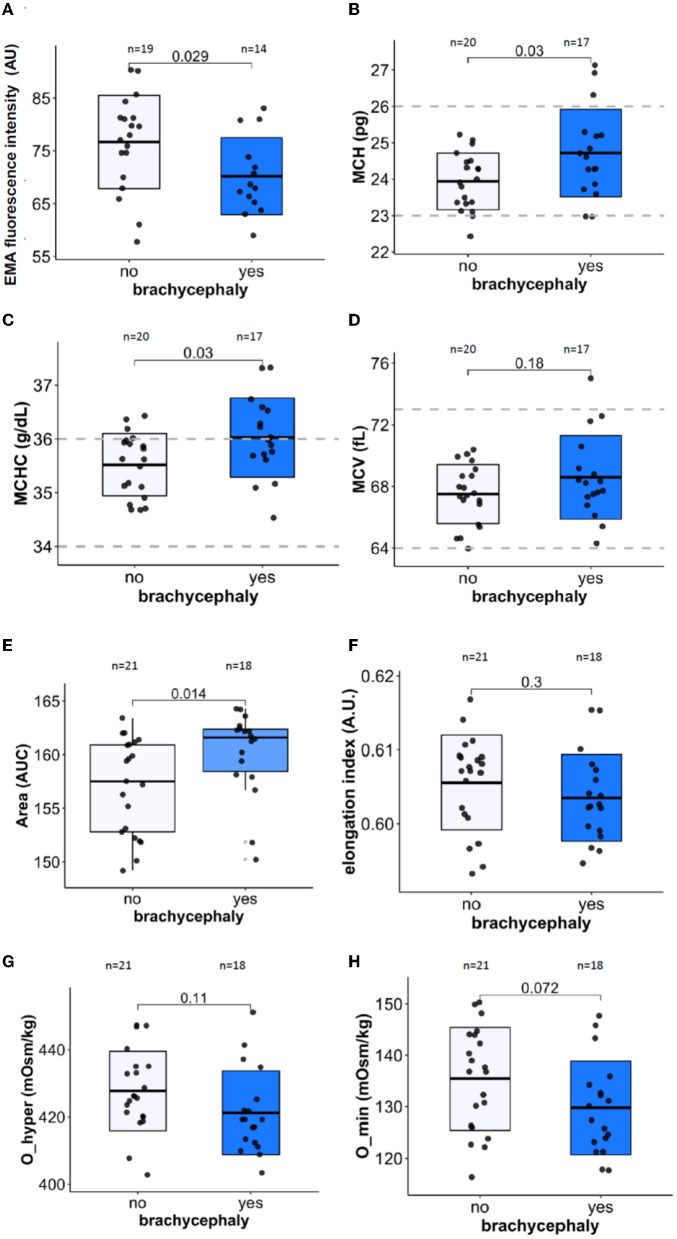
Membrane stability, RBC indices, cell deformability, and hydration markers. Membrane surface area using EMA staining (band 3 protein) normalized to human control **(A)**, Mean cell hemoglobin [MCH, **(B)**], Mean cell hemoglobin concentration [MCHC, **(C)**], and Mean cell volume [MCV, **(D)**]. The LoRRca osmoscan mode was used to detect the following ektacytometry indices: Area [the range of osmolarities tolerated while able to deform, **(E)**], Maximal elongation index EImax [deformability, **(F)**], Maximal tolerated osmolarity O_hyper [hydration state of RBCs, **(G)**], and minimal tolerated osmolarity O_min [osmotic stability, **(H)**]. Unpaired Student's *t*-test was performed on the datasets except for the Area parameter. For the latter, Wilcoxon rank sum test was performed.

RBCs of the BC group were presented with facilitated membrane loss that was visualized as lower band 3 protein abundance using EMA test (Figure 3A). Furthermore, mean corpuscular Hb (MCH, Figure 3B) and mean corpuscular Hb concentration (MCHC, Figure 3C) were significantly higher in the BC dogs compared to the NC dogs. Mean cell volume (MCV, Figure 3D) did not differ between the two groups.

Further information on the RBC hydration, membrane stability, and deformability was obtained using ektacytometry. The osmoscan curves for the blood samples of the BC group was shifted to the left compared to those of the NC dogs. The area under the osmoscan curve for the BC group significantly exceeded that for the NC RBCs (Figure 3E). “Area” was the only parameter in our study that depended on the neutering status of the dogs (*p* = 0.008) in both BC and NC groups, while all the other parameters measured did not show any association with sex, age, or neutering state. Elongation index reflecting RBC deformability (EImax, Figure 3F) and the lowest tolerable osmolarity O_min associated with osmotic stability (Figure 3H) did not significantly differ between the two groups. In agreement with an increase in MCHC, RBCs of BC group were less tolerant to hyperosmotic stress than the RBCs of the NC dogs (Figure 3G).

### 3.4. RBC redox state and inflammatory and stress markers

**Figure 4:**
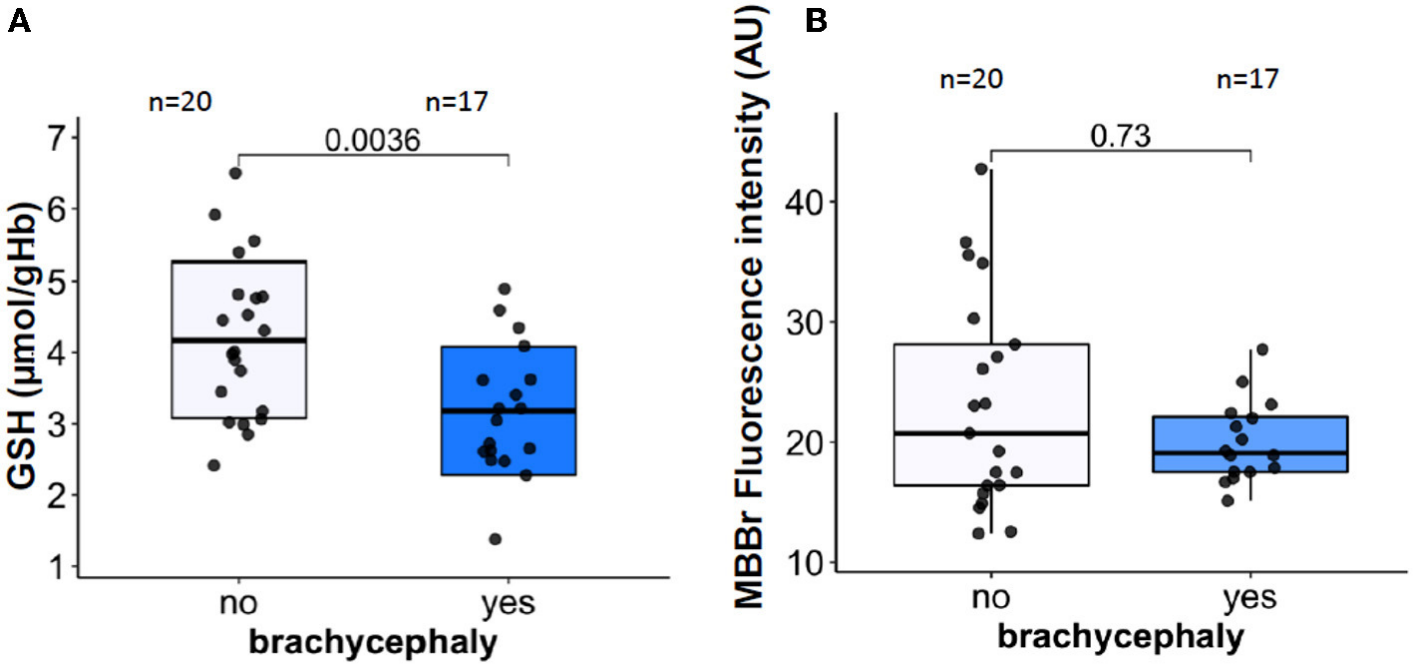
RBC oxidation markers. Intraerythrocytic non-protein reduced thiols represented mainly by reduced glutathione [GSH, **(A)**], and total (non-protein and protein) reduced thiols assessed as monobromobimane (MBBr) fluorescence intensity **(B)**.

In blood samples that were analyzed within 30 min after blood collection, intraerythrocytic GSH for the BC group was significantly lower than that for the NC dogs (Figure 4A). The observed difference in GSH was not mirrored by the reduction in total (protein and non-protein) thiols that were detected as MBBr fluorescence (Figure 4B). No difference in met-Hb levels was detected between BC and NC dogs (see raw dataset).

**Figure 5:**
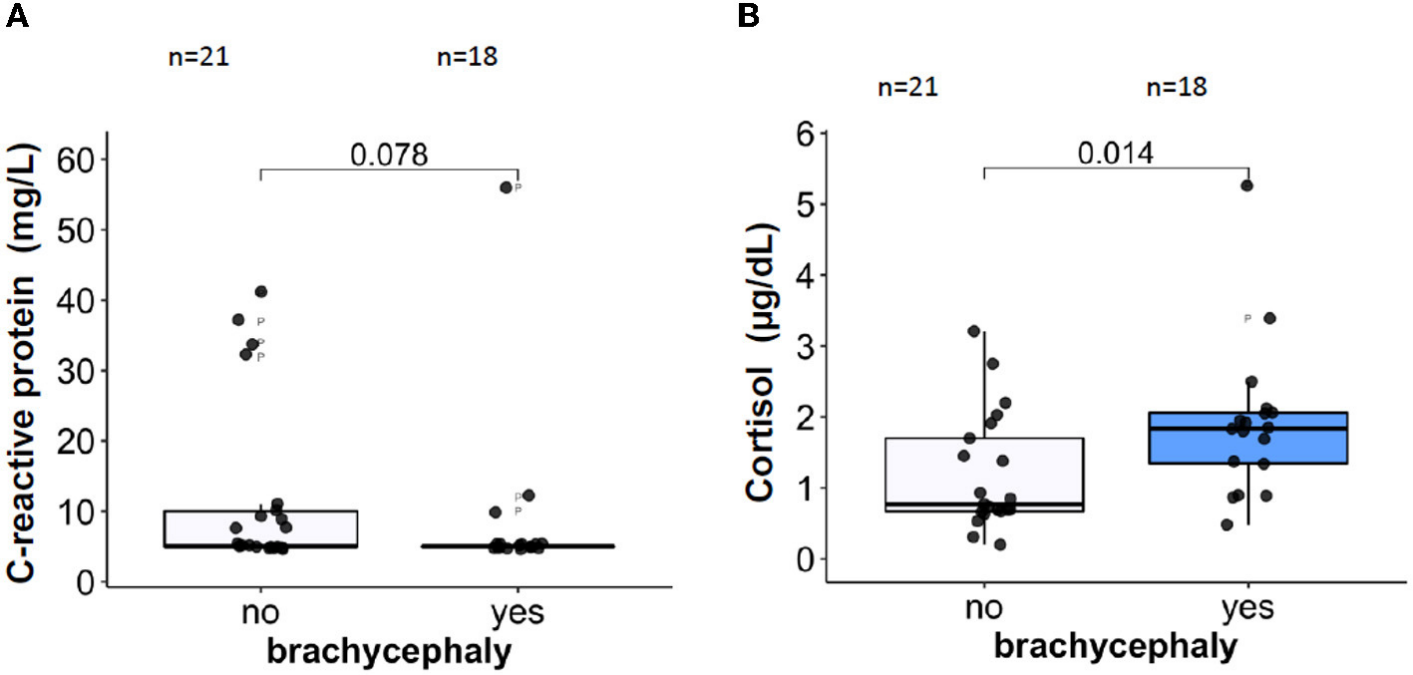
Plasma concentrations of the inflammatory marker C-reactive protein CRP, **(A)** and of a stress marker, cortisol **(B)**.

Plasma CRP, an acute phase inflammation marker, was not different in the BC dogs compared to the NC dogs (Figure 5A). For some dogs from both groups, the plasma CRP values exceeded the upper reference value of 10.7 mg/L (46). The majority of the blood samples with pathologically high CRP levels originated from NC dogs. Plasma concentrations of a stress marker cortisol, were significantly higher in the BC group compared to the NC group (Figure 5B). The median cortisol concentrations in the BC group were approaching the upper reference threshold of 26 μg/L (65). Total leukocyte and absolute neutrophil counts did not differ between the two groups (Table 2). However, absolute lymphocyte and eosinophil counts were significantly lower in the BC dogs compared to the NC dogs (Table 2). No difference was found between the two groups in monocyte, basophil, and platelet counts or in thrombocrit (Table 2).

**Table 2:** Total white blood cell differential and platelet counts in venous blood samples of non-brachycephalic (*n* = 22) and brachycephalic dogs (*n* = 18).

**Parameter**	**Reference**	BC group Median^*^/mean	Lower-upper IQR^*^or SD	***N***	NC group Median^*^/mean	Lower-upper IQR^*^SD	***N***	***p***
Leukocytes, x10E3/μL	4.74–11.3	8.52	5.33–10.71	18	8.62^*^	7.57–11.22	22	0.355
Neutrophils, x10E9/L	2.54–7.44	5.39	3.52–6.73	18	5.01^*^	3.98–5.92	22	0.87
Lymphocytes, x10E9/L	1.154–3.4	2.09	0.99	18	3.08	1.38	22	**0.015**
Neutrophil/ lymphocyte ratio		2.63	1.75–4.85	18	1.72	1.15–2.43	22	**0.009**
Eosinophils, x10E9/L	0.124–1.29	0.23	0.16–0.48	18	0.58^*^	0.40–0.89	22	**0.02**
Basophils, x10E9/L	04–0.08	0.02	0.007–0.022	18	0.02^*^	0.010–0.02	22	0.93
Monocytes, x10E9/L	0.24–0.92	0.59	0.35	18	0.68	0.24	22	0.35
Platelets, x10E9/L	1304–394	555	340–751	17	456	307–564	22	0.15
Thrombocrit %	0.14–0.61	0.29	0.17–0.41	17	0.24	0.19–0.31	22	0.24

Normality Shapiro–Wilk test performed. If the distribution was proven normal, it was followed by a Student's t-test statistics (means and SD shown). If normality test was failing, ^*^Mann–Whitney rank sum test was performed. Shown in the table are the medians and the lower-upper interquartile ranges (IQR). P values below 0.05 signifying the statistically significant differences between the BC and NC groups are highlighted in bold.

### 3.5. Metabolic changes

**Figure 6:**
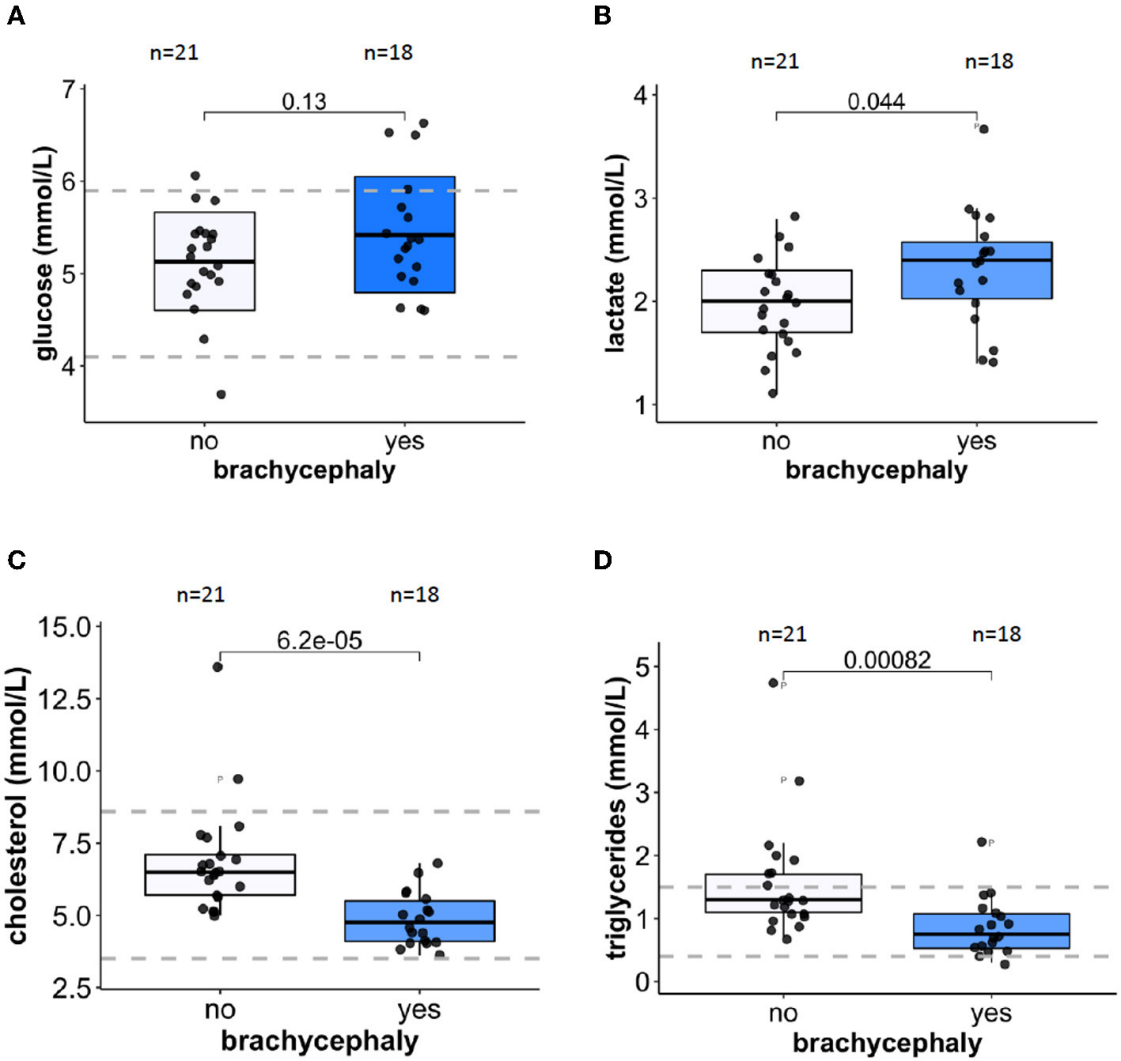
Metabolic markers in plasma. Glucose **(A)** and lactate **(B)** concentrations in plasma of 21 NC and 18 BC dogs. Plasma concentrations of cholesterol **(C)** and triglycerides **(D)** for 21 NC and 18 BC dogs. Dotted lines indicate healthy reference range.

Plasma glucose concentrations were similar for both groups (Figure 6A) but plasma lactate levels were significantly higher in BC dogs compared to NC dogs (Figure 6B). Plasma cholesterol (Figure 6C) and triglyceride (Figure 6D) concentrations were significantly lower in the BC blood samples compared to the NC dogs. When normalized to blood Hb levels, lactate production rate during the simulated transportation did not differ between the BC and NC groups.

### 3.6. Outcome of the simulated transportation study (part II)

**Figure 7:**
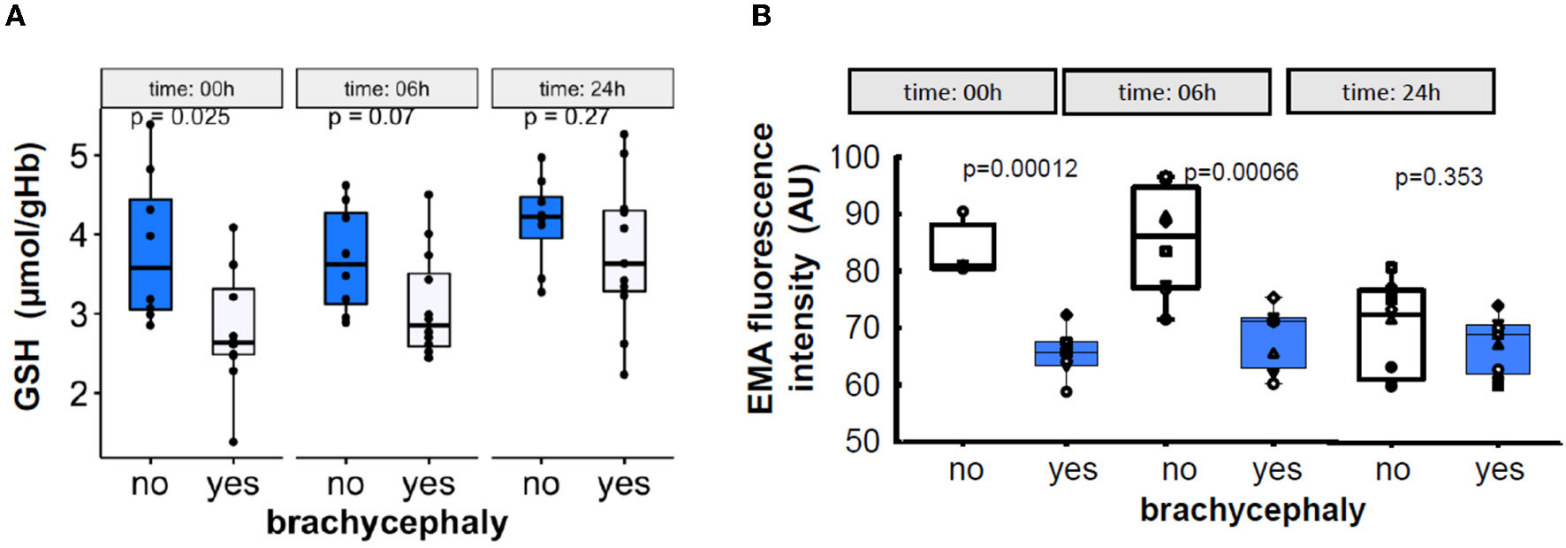
Time-dependent alterations in the intraerythrocytic GSH levels **(A)**, and in the abundance of band 3 protein in RBC membrane measured as EMA fluorescence intensity **(B)** during the 24 h simulated transportation.

Simulated “transportation” of the blood samples was associated with a gradual recovery of intracellular GSH levels in RBCs of the BC group that did not differ from those in the NC group after the 6 h of the “shipment” (Figure 7A). Furthermore, RBCs of the NC dogs shed some of the membrane which contained band 3 protein. As a result, a 24 h of shipment resulted in reduction in EMA staining intensity of the RBCs in the NC group which then did not differ from the values obtained for the RBCs of the BC dogs (Figure 7B).

## 4. Discussion

The obtained data reveal the existence of a particular pattern of blood parameters associated brachycephaly. We were unable to confirm reduced blood oxygenation of BC dogs directly by monitoring hemoglobin oxygen saturation (Figure 1D). We also failed to observe signs of polycythemia i.e., an increase in erythrocyte count in the BC dog group (Table 1). In 1.6–10% of human patients with OSA that spend more time with SaO_2_ lower than 90%, polycythemia was reported (66). Still, the modest increase in reticulocyte count (Figure 1B) and a higher abundance of MFI reticulocytes (Figure 1C) may be indicative of stress erythropoiesis. These features were also reported for patients with OSA, for whom a strong association between the higher abundance of immature reticulocytes with duration of hypoxic phases during sleep was shown (67). Marrone et al. (67) reported that abundance of MFI reticulocytes shows maximal association with the apnea/hypopnea index, and with the lowest arterial O_2_ saturation, while the HFI reticulocyte counts do not correlate with these parameters. In human subjects with OSA, reduction in reticulocyte maturation was not accompanied by an increase in plasma erythropoietin levels. This resembles an early increase in immature reticulocytes in heathy subjects acutely exposed to high altitude hypoxia (3500 m above sea level) observed at the day of assent before an increase in plasma erythropoietin could be detected (68). Washout of the pre-existing immature reticulocytes from bone marrow compartment rather than an increase in *de novo* production of reticulocytes in response to acute hypoxic challenge was suggested as a mechanism for this effect.

RDW was the most relevant variable of all RBC indices that distinguished NC from BC groups (Supplementary Figures 1–3). Upregulation of RDW, which was shown to be a non-specific indicator of distress in humans (69, 70) and in dogs (71–73), was observed for the BC group. However, RDW remained below the upper reference threshold for healthy animals for both groups (Figure 2A). An increase in RDW in the BC group was not associated with parameters indicative for facilitated RBC destruction such as an increase in plasma bilirubin of CO-Hb levels. However, higher reticulocyte counts in BC dogs suggest faster RBC turnover in this group (Figure 1B). Alternatively, the “washout” effect mentioned above may be the cause. Measurements of plasma erythropoietin concentrations that were not performed in this study could add to the understanding of the possible causes of higher RDW in BC group.

Our data reflected higher levels of RNO in the plasma of BC dogs compared to the NC group (Figure 1A). NO release in response to acute or chronic hypoxia induces vasodilatation in humans (74) and dogs (75, 76). The pattern of changes in NO production during chronic intermittent hypoxia is more complex. Airway NO production is proportional to the OSA severity grade in human patients (77). Plasma NO bioavailability is reduced in OSA patients due to the oxidative stress. An increase in asymmetric dimethylarginine (ADMA) levels that trigger uncoupling of NO synthases further diminishes plasma NO content (78, 79) as superoxide anion-induced derivatization of NO to ONOO^−^ decreases the NO levels even further (80). In contrast to that of OSA-affected human patients, intermittent hypoxia in BC dogs is associated with upregulation of RNOs (Figure 2B) (36). This feature may represent breeding-associated genetic or epigenetic adaptation as increased NO bioavailability supports survival under hypoxic conditions in humans (81, 82) and animals (83). Endothelial NO synthase gene expression is under the control of promoter-containing hypoxia-responsive elements (84). Earlier studies revealed that plasma levels of NO derivatives in dogs are breed-dependent, being higher in Cairn Terriers than in Pointers, and the lowest in sled dogs (Alaskan huskies mixed with German and English Pointer) or in King Charles Spaniels (85). In the same study, the effect of exercise on RNO in sled dogs was shown, and the need for gender-stratification was mentioned. Thus, the use of RNO as an indicator of intermittent hypoxia in dogs still must be elucidated.

Inflammatory state is reported for pathological conditions resulting from intermittent hypoxia in humans with OSA (43, 86, 87) and dogs presenting with BOAS (12, 37, 39). The missing differences in the CRP concentrations between BC and NC dogs in our study may reflect differences in study designs. In the present study we compared blood parameters of non-brachycephalic with those of brachycephalic dogs. In the other studies two groups of brachycephalic dogs, one with and one without BOAS, were compared with each other. An upregulated neutrophil-to-lymphocyte ratio (Table 2) as well as the increase in plasma cortisol levels (Figure 5B) that we observed in the blood of the BC group have been recognized as stress response markers in a number of species, including dogs (88). Recently, similar changes in leukogram were reported for dogs challenged with short-distance road transport (89). In humans, a similar increase in neutrophil-to-lymphocyte ratio was observed for the severe OSA state (90).

Despite the lack of inflammation, RBCs of BC dogs showed signs of oxidation (Figure 5A). Mature canine RBCs lack mitochondria but the other peripheral cell types do have them. Production of reactive oxygen species by hypoxic mitochondria was shown for endothelial cells, myocardium, and the brain (91). Reoxygenation adds to the free radical production that is persisting in the tissues of patients with OSA exposed to hypoxia-reoxygenation bouts (92). The ability of the RBCs of the BC dogs to restore GSH levels extracorporeally during the simulated transportation (Figure 7B) suggests that the oxidative load causing a drop in GSH most likely reflects production of oxidants by the peripheral tissues as in humans with OSA (92, 93). Interestingly, whole-blood superoxide dismutase (SOD) activity was found to be decreased in dogs with severe BOAS (grade 2–3) before corrective surgery (94), although it recovered within 2 weeks after surgery. As SOD in blood is mainly represented by the pool located in RBCs; this fast recovery indicates that the suppression of SOD activity was reversible. Canine RBC life-span is about 110 days (95), and most of the RBCs circulating 14 days after the surgery were produced before the BOAS correction was performed. RBCs are not able to replace damaged and permanently inactivated proteins, thus the partial inactivation of SOD was reversed gradually after the operation as RBCs were replaced by newly produced ones. Of note, glutathione peroxidase activity in whole blood and malondialdehyde levels in plasma of BOAS-affected brachycephalic dogs did not differ from that in brachycephalic dogs without the signs of BOAS (94). Additional investigation of peroxiredoxins and catalase activity could provide further insights on the impact of BOAS on the antioxidant capacity of RBCs.

The impact of OSA on RBC shape and membrane surface:volume ratio has been documented due to vesiculation (96). In human RBCs, vesicle formation is promoted by Ca^2+^ uptake (97) and oxidative stress (98), and rheology of RBC gets affected by the membrane loss (99). Although we could not confirm statistically significant alterations in RBC deformability in BC dogs, the tendencies to RBC dehydration were observed for these dogs (Figures 3F, G). Furthermore, facilitated vesiculation and loss of band 3 protein as follows from the EMA test (Figure 3A) contributes to heterogeneity of RBC shapes and sizes, which is reflected by the increase in RDW (Figure 2A). This parameter proved to be the best predictor of BC phenotype amongst all the RBC indices (Figure 2A and Supplementary Figures 1–3). The currently accepted “healthy” reference range for RDW for dogs is rather broad and is not stratified for breeds. The obtained data, however, do not provide evidence for the possible association of RDW with BOAS severity. RDW is a non-selective parameter reflecting distress in dogs with critical illness, and predicting severity of disease state and mortality prognosis in dogs (72, 100) and in humans (101). Vesiculation and membrane loss and changes in morphology of RBCs contribute to the increase in variance of RBCs that are exposed to repetitive shear stress (102).

Metabolic consequences of intermittent hypoxia include insulin resistance and obesity in humans (103, 104), resulting from the stabilization of the hypoxia-inducible transcription factor 1-α (HIF1-α), and from increasing sympathetic neural activity that is crucial for the regulation of glucose and fat metabolism (104). We have observed a difference in parameters reflecting glucose and lipid metabolism between the BC and NC dogs. Plasma glucose concentrations did not differ between the groups in our study (Figure 6A). Earlier on, slightly higher glucose levels reported in BC dogs were associated with the severity of anatomical abnormalities (39). Plasma of BC dogs in our study contained slightly but significantly more lactate that that of NC dogs (Figure 6B). For both groups the absolute values were within the “healthy” reference range for dogs [0.3–2.5 mM (105)]. This may be an indirect indication of transient recurrent hyperglycemia associated with hypoxic bouts for Pugs, French Bulldogs, and English Bulldogs as reported by Gianella et al. (39). Plasma lactate concentrations reflect its cumulative production by RBCs and peripheral tissues. As we could not confirm the BC-to-NC differences in lactate production by RBCs in a simulated transportation study (data not shown), this minor increase in plasma lactate of BC dogs may be caused by facilitated glycolysis of the peripheral tissues. Lactatemia detected within 24 h after birth was shown to be a negative prognostic indicator for survival for puppies of brachycephalic breeds (38).

In our study we have observed lower cholesterol and triglyceride levels in plasma of BC dogs compared to NC ones (Figures 6C, D). These unexpected observations were not in line with the data reported for rodents and humans exposed to chronic continuous or short-term intermittent hypoxia. While in rodent models a decrease in oxygenation was always associated with an upregulation of plasma cholesterol and triglyceride levels, contradictory findings (from an increase to no response) were reported in several human studies (106, 107). Measurements of plasma cholesterol and triglyceride levels in brachycephalic dogs with various degrees of BOAS severity did not reveal a significant association between these parameters and severity of the pathological condition (39). In our BC group, plasma cholesterol and triglyceride levels in the BC group were on average lower than those in the NC group (Figures 6C, D) but still within the reference range for healthy animals.

This study was performed to identify the blood parameters that show dependence on the cranial anatomical features typical for brachycephaly. Based on our findings, the parameters of interest include the RNO, GSH, RBC indices (particularly the RDW), reticulocyte counts and their maturity, stress markers (cortisol and leucocytes-to-neutrophils ratio), cholesterol, and triglycerides. Using breed lists to categorize brachycephalic and non-brachycephalic dogs may limit the explanatory power of our study, as dogs assigned to mesocephalic breeds such as Pomeranians may also suffer from small dimensioned rostral airways without the owners being aware of it. The severity of brachycephalic state and the sensitivity and specificity of the chosen parameters will be tested in a follow-up study.

## Data availability statement

The datasets presented in this study can be found in online repositories. The names of the repository/repositories and accession number(s) can be found in the article/Supplementary material.

## Ethics statement

The animal study was reviewed and approved by the Veterinary Department of the Canton Zurich (#ZH161/19). Written informed consent was obtained from the owners for the participation of their animals in this study.

## Author contributions

IR, AB, and RH-L planned the study and received funding for it. IR recruited the study participants and collected blood. SK, SF, AV, NB, and AB performed experiments and analyzed blood samples. SK, SF, AB, and SH analyzed the data. AB, SK, IR, RH-L, and MS produced a manuscript draft. All authors contributed to the article and approved the submitted version.
