# Supplementary material for: Differences in selected blood parameters between brachycephalic and non-brachycephalic dogs
Source: Front Vet Sci. 2023 Aug 15;10:1166032. doi: 10.3389/fvets.2023.1166032 (PMC10464621; doi:10.3389/fvets.2023.1166032)

Supplementary Information for the article Kämpf et al., (2023) Differences in selected blood parameters between brachycephalic and non-brachycephalic dogs. Front. Vet. Sci. 10:1166032. doi: 10.3389/fvets.2023.1166032

Supplementary Table S1 Description of the study cohort

**Nonbrachycephalic group (22 dogs)**					
**Dog ID**	**Breed**	**Age, years**	**Sex**	**Included in part I**	**Included in part II**
1	Golden Retriever	6	female*	Yes	No
2	Jack Russel Terrier	2.5	male	Yes	No
3	Barsoi	8.5	female	Yes	No
4	Border Collie	6.5	male	Yes	No
5	Labradoodle	0.5	male	Yes	No
6	Spitz mix	11	female*	Yes	No
8	Terrier mix	1.5	female*	Yes	No
12	Scent Hound mix	7	female*	Yes	No
13	Carpathian Shepherd Dog	3	female*	Yes	No
16	Cattle Dog mix	8	female*	Yes	No
17	Spitz mix	3	female*	Yes	No
18	Pomeranian	1	female	Yes	No
19	Bolognese Dog	12.5	male*	Yes	No
20	Labrador Retriever	6	female*	Yes	No
24	Beauceron	2	female	No	Yes

25	Smooth Collie	1	female	No	Yes
26	Dobermann	6	male	No	Yes
30	German Shepherd mix	1	female	No	Yes
31	Scent Hound mix	5	female	No	Yes
32	Icelandic Sheepdog	7	female	No	Yes
33	Kelpie	1.5	female	No	Yes
34	Rottweiler	7.5	female*	No	Yes

**Brachycephalic group (18 dogs)**					
**Dog ID**	**Breed**	**Age, years**	**Sex**	**Included in part I**	**Included in part II**
7	French Bulldog	10	female*	Yes	No
9	French Bulldog	12	female	Yes	No
10	English Bulldog	1.5	female	Yes	No
11	Pug	12	male	Yes	No
14	Pug mix	0.5	male*	Yes	No
15	Pug mix	10	female	Yes	No
21	French Bulldog	6.5	female	No	Yes
22	French Bulldog	6.5	female*	No	Yes
23	French Bulldog	4	female*	No	Yes
28	Bulldog mix	3	female*	No	Yes

29	Continental Bulldog	4	female*	No	Yes
35	French Bulldog	7	female	No	Yes
36	French Bulldog	11	female	No	Yes
37	French Bulldog	9	female	No	Yes
38	French Bulldog	1	male	No	Yes
39	French Bulldog	0.5	male	No	Yes
41	French Bulldog	1	male	No	Yes
42	Pug	10	female	No	Yes

Breed, age, sex and reproductive status of the dogs included in the non-brachycephalic (NC) and brachycephalic group (BC). Part I: evaluation study, Part II: transportation study. For details see the text. Sex: *= spayed/castrated

Figure s1

Visual assessment of the varrank output indicating ranking of the seven variables of red blood indices in distinguishing NC and BC dogs.

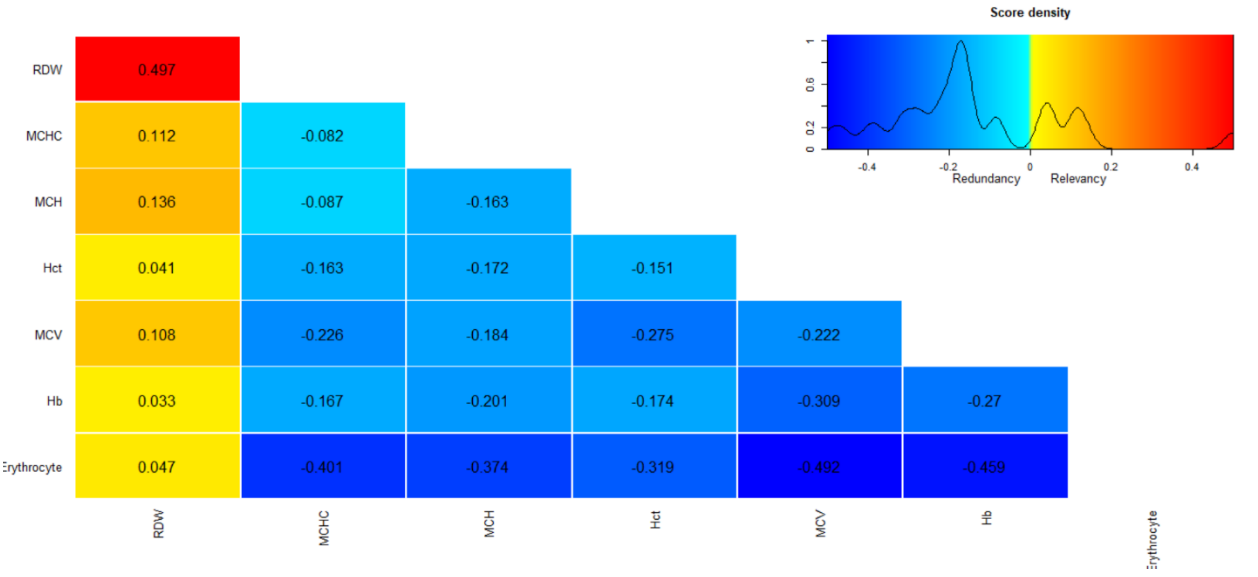

The color in the first column indicates the relevancy of each variable with hot red for higher relevancy and in the other columns blue for redundancy with darker blue for higher redundancy. The numbers represent the scores indicating the importance of the variable to predict NC or BC.

Figure s2

caret package

Output from the caret package performing a model-free selection search. Based on ROC the seven variables of red blood indices are ordered according to their importance for predicting NC or BC dogs.

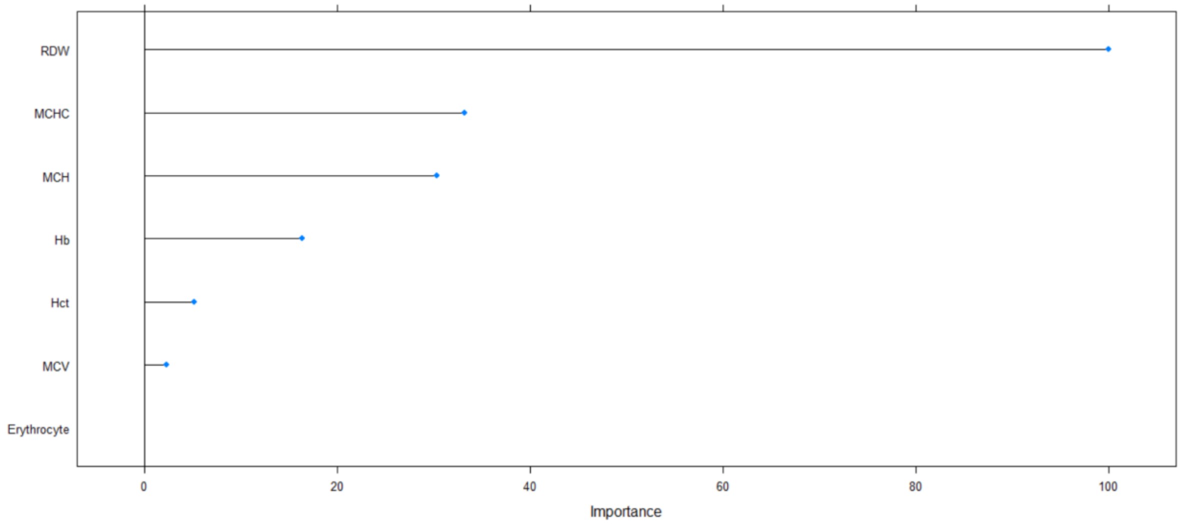

Figure s3

Boruta package

Based on a random forest approach with the Boruta package (CIT), the variables are ordered according to their importance in predicting NC or BC dogs.

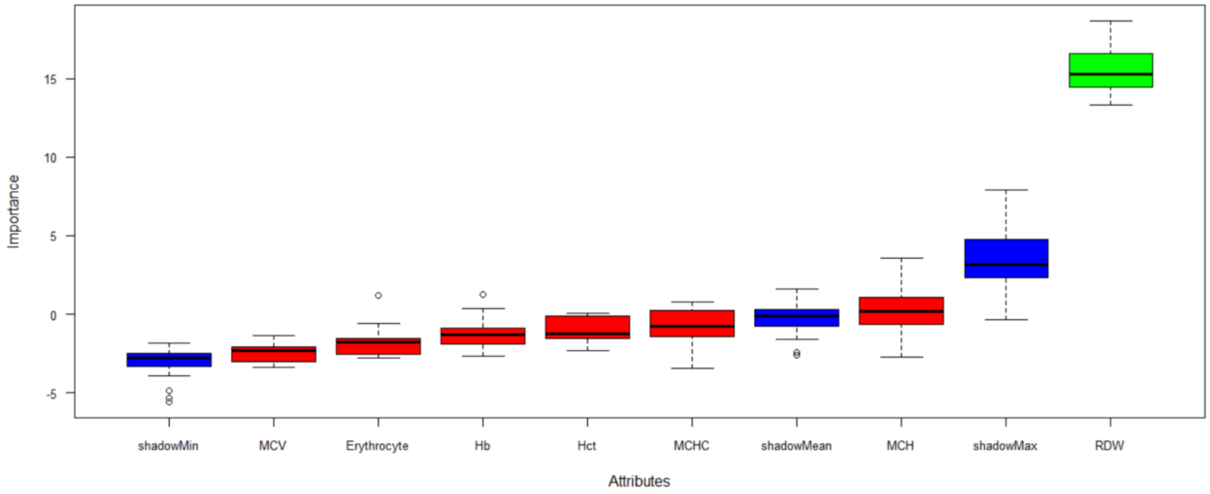

Supplement: Supplementary file 1 [file Data_Sheet_1.pdf]
